# Association of N-Terminal Pro-brain Natriuretic Peptide With Volume Status and Cardiac Function in Hemodialysis Patients

**DOI:** 10.3389/fcvm.2021.646402

**Published:** 2021-02-22

**Authors:** Yaqiong Wang, Xuesen Cao, Jinbo Yu, Yongmei Zhang, Xianzhe Li, Xiaohong Chen, Jianzhou Zou, Bo Shen, Xiaoqiang Ding

**Affiliations:** ^1^Department of Nephrology, Zhongshan Hospital, Fudan University, Shanghai, China; ^2^Shanghai Medical Center of Kidney, Shanghai, China; ^3^Department of Nephrology, Shanghai Institute of Kidney and Dialysis, Shanghai, China; ^4^Shanghai Key Laboratory of Kidney and Blood Purification, Shanghai, China; ^5^Hemodialysis Quality Control Center of Shanghai, Shanghai, China

**Keywords:** volume overload, N-terminal pro-brain natriuretic peptide, body composition monitor, ejection fraction, hemodialysis

## Abstract

**Introduction:** N-terminal-pro-brain natriuretic peptide (NT-pro BNP) is secreted by cardiomyocytes in cases of cardiac structure disorder and volume overload. However, the relationship between NT-pro BNP level and body fluid status in dialysis patients with reduced cardiac ejection function (EF) is uncertain. Therefore, we aimed to investigate this relationship.

**Methods:** We enrolled patients who had been receiving hemodialysis for >3 months. Blood sample, transthoracic echocardiographic, and bioimpedance spectroscopy measurements were performed during a midweek non-dialysis day. The predictive value of NT-pro BNP in hemodialysis patients with volume overload was analyzed.

**Results:** A total of 129 hemodialysis patients (74 men and 55 women; mean age: 59.4 ± 13.0 years) were recruited. The average hemodialysis duration was 55.5 (23.9–93.4) months, the NT-pro BNP level was 4992 (2,033–15,807) pg/mL, and the value of overhydration was 2.68 ± 0.19 (−1.9 to 12.2) L. The NT-pro BNP level was independently correlated with overhydration in both the LVEF ≥ 60% (β = 0.236, *P* = 0.044) and LVEF <60% (β = 0.516, *P* = 0.032) groups, even after adjustments for potentially confounding variables. In receiver operating characteristic curves of NT-pro BNP for predicting volume overload, the area under the curve was 0.783 [95% CI (0.688–0.879), *P* < 0.001) and 0.788 [95% CI (0.586–0.989), *P* < 0.001] in the LVEF ≥ 60% and LVEF < 60% groups, respectively.

**Conclusions:** NT-pro BNP is a predictive factor for volume overload in hemodialysis patients with or without EF declines.

## Introduction

Overhydration has been identified as an important predictor of mortality in chronic kidney disease (CKD) and hemodialysis patients (1–3). Volume overload is associated with systemic hypertension, left atrial dilation, left ventricular hypertrophy, inflammation, malnutrition, and the eventual development of congestive heart failure (4, 5). Excessive or rapid reduction in circulating blood volume causes intradialytic hypotension, adverse symptoms, and end-organ hypoperfusion (6, 7). Thus, it is important to appropriately address volume status in dialysis patients in order to reduce the incidence of hypertension and cardiovascular mortality in patients.

Volume management in hemodialysis patients is dependent on the availability of accurate and objective methods for assessing volume status. Clinical examination is currently the mainstay of volume assessment; however, this approach is imprecise and unreliable. Other methods include evaluation of the inferior vena cava diameter with ultrasound, radionuclide dilution techniques, and echocardiography, however, these are time-consuming and difficult to perform (8).

As an alternative, the bioimpedance spectroscopic method has been used to evaluate hydration status. Its accuracy has been validated by isotope dilution methods (9). Based on the bioimpedance principle, the Body Composition Monitor (BCM; Fresenius Medical Care, Bad Homburg, Germany), can measure the patient's body composition and hydration status (10); this may be used for fluid management in order to control blood pressure and decrease arterial stiffness (11–13).

Brain natriuretic peptide (BNP) and N-terminal pro-BNP (NT-pro BNP) have been reported to be associated with volume status in several studies (14, 15). BNP is a polypeptide that is secreted by cardiomyocytes in response to hypoxia and excessive stretching. Pre-pro BNP is transformed into pro BNP, which is subsequently split into BNP and the biologically stable NT-pro BNP. The higher serum concentration of NT-pro BNP can be attributed to its longer half-life of 1 to 2 h compared to BNP's half-life of 20 min (16). NT-pro BNP and BNP have been associated with cardiac dysfunction and volume overload. However, BNPs levels may be affected by the dialysis procedure, serum creatinine concentration, and cardiac disorders which may affect its role as a marker of overhydration in patients undergoing dialysis (17–19). Moreover, it remains unknown whether NT-pro BNP can serve as a predictor of volume status in dialysis patients with cardiac dysfunction. In this study, we aimed to assess the relationship of NT-pro BNP with volume status, with or without cardiac dysfunction, in hemodialysis patients using the bioimpedance spectroscopic method.

## Materials and Methods

### Study Population

In this cross-sectional study, 129 hemodialysis patients, who had been receiving hemodialysis treatment for more than 3 months, were enrolled from the Blood Purification Center, Zhongshan Hospital, Fudan University. Exclusion criteria included patients who had experienced angina pectoris, acute myocardial infarction, cerebral infarction, and cerebral hemorrhage within 3 months before the study or those who were <18 years old. The study complied with the Declaration of Helsinki and was approved by the Ethical Committee, Zhongshan Hospital, Fudan University. All participants provided written informed consent.

Patients were treated three times per week and 4 h per session with the standard bicarbonate dialysate by low-flux hemodialysis using 1.4 m^2^ dialyzers with synthetic membranes (BLS514SD, Sorin Group Italia, Mirandola, Italy; Polyflux 14L, Gambro Dialysatoren GmbH, Hechigen, Germany). The blood flow was 200–280 mL/min, and the dialysate flow rate was 500 mL/min.

### Data Collection and Biochemical Measurements

Height and weight were measured before the patients' hydration status was assessed using the BCM. Demographic and clinical data were collected, including age, sex, primary kidney disease, dialysis duration, comorbidity, and current medications.

Blood sampling was performed during a midweek non-dialysis day from 8 to 10 a.m. after a 30 min rest in a semi-recumbent position. Hemoglobin (Hb), serum albumin (Alb), serum creatinine (SCr), uric acid (UA), calcium (Ca), phosphorus (P), and ferritin were measured using standard procedure. The concentrations of β2-microglobulin and intact parathyroid hormone were determined using an immunoturbidimetry assay and electrochemiluminescence immunoassay, respectively.

### Echocardiography

Transthoracic echocardiographic examinations were performed by a single experienced cardiologist using a Philips echocardiographic machine (Philips IE33; Philips, Eindhoven, The Netherlands) within 2 h after blood sampling. Left atrial diameter (LAD), left ventricular end diastolic diameter (LVEDD), left ventricular end systolic diameter (LVESD), left ventricular posterior wall thickness (PWT), interventricular septum thickness, and left ventricular ejection fraction (LVEF) were measured. Left ventricular mass (LVM) was calculated using the Devereux Equation (20), and the left ventricular mass index (LVMI) was the ratio of LVM to body surface area.

### Assessment of Body Composition

On the same day, hydration status was measured using the BCM. Blood pressure and heart rate were obtained before BCM examination. Body composition parameters such as overhydration (OH), total body water (TBW), extracellular water (ECW), intracellular water (ICW), the ratio of ECW to ICW (E/I), and relative overhydration (ROH, ROH = OH/ECW) were recorded. Volume overload was defined as ROH ≥ 15% (in men) and ≥13% (in women) (1).

### Statistical Analysis

Numerical variables are expressed as means ± SDs and medians (interquartile ranges). Because the level of NT-Pro BNP does not follow the normal distribution, we use the lg transformation in the statistical process. Comparison between groups was performed by ANOVA, Mann–Whitney U and chi-squared tests. A two-tailed *P*-value of < 0.05 was considered statistically significant. All analyses were performed using SPSS 20.0 (SPSS Inc., Chicago, IL, USA).

## Results

### Patients' Characteristics

A total of 129 hemodialysis patients (74 men and 55 women) were recruited {mean age [median (interquartile range)]: 59.4 ± 13.0 [21–88]} years. The dialysis duration was 55.5 (23.9–93.4) months. The primary end-stage renal diseases were chronic glomerulonephritis (27.6%), diabetic nephropathy (15.3%), hypertensive nephropathy (2.4%), and polycystic kidney disease (8.8%). Among the patients, the level of NT-pro BNP was 4,992 pg/mL (2,033–15,807) and the value of overhydration was 2.68 ± 0.19 (−1.9 to 12.2) L. Patients with LVEF <60% accounted for 21.5% of the cohort and the incidence rate of volume overload was 53.9%.

The main clinical characteristics of the patients across the quartiles of NT-pro BNP are shown in Table 1. Compared with the lower quartile NT-pro BNP group, the higher quartile NT-pro BNP group had lower Hb (*P* < 0.001), HCT (*P* = 0.003), and Alb (*P* = 0.009) and higher SBP (*P* = 0.005) and volume status parameters, such as OH (*P* < 0.001), ROH (*P* < 0.001), and E/I (*P* < 0.001). However, patients in the higher quartile NT-pro BNP group had higher LAD (*P* < 0.001), LVEDD (*P* = 0.023), LVESD (*P* < 0.001), PWT (*P* = 0.049), and LVMI (*P* < 0.001) and lower LVEF (*P* < 0.001).

**Table 1:** Baseline clinical and biochemical characteristics of 129 patients on hemodialysis.

**Characteristic**	**NT-pro BNP ≤ 2,033 (*n* = 32)**	**2,033 < NT-pro BNP ≤ 4,992 (*n* = 32)**	**4,992 < NT-pro BNP ≤ 15,807 (*n* = 33)**	**15,807 < NT-pro BNP ≤ 35,000 (*n* = 32)**	***P*-value**
Age (years)	58.2 ± 11.6	59.9 ± 13.9	62.4 ± 13.6	59.9 ± 12.4	0.681
Sex (men, %)	62.1	62.1	32.1	69.0	0.024
HD Durations (months)	47.7 (21.1–96.1)	67.9 (30.2–108.3)	48.8 (31.7–92.3)	53.9 (25.5–74.9)	0.196
BMI (Kg/m^2^)	23.2 ± 2.8	22.7 ± 4.2	22.2 ± 3.9	22.3 ± 3.6	0.710
BSA (m^2^)	1.69 ± 0.2	1.68 ± 0.2	1.57 ± 0.2	1.69 ± 0.2	0.036
SBP (mmHg)	136 ± 20	140 ± 25	144 ± 22	158 ± 20	0.005
DBP (mmHg)	77 ± 11	78 ± 17	78 ± 11	82 ± 20	0.701
MAP (mmHg)	97 ± 13	99 ± 19	100 ± 13	107 ± 17	0.113
HR (bpm)	79 ± 10	75 ± 10	74 ± 10	75 ± 11	0.281
**Laboratory parameters**
Hb (g/L)	119 ± 11	113 ± 14	110 ± 12	101 ± 21	<0.001
HCT (%)	38 ± 4	36 ± 5	35 ± 4	32 ± 7	0.003
Alb (g/L)	41 ± 3	40 ± 3	39 ± 3	37 ± 8	0.009
Cr (μmol/L)	1,025 ± 317	1,051 ± 295	860 ± 239	965 ± 285	0.058
β_2_MB (mg/L)	31.8 ± 9.8	36.6 ± 7.4	33.1 ± 10.5	36.2 ± 9.9	0.155
iPTH (pg/mL)	226.5 (155.8–319.0)	258.0 (166.0–487.5)	214.0 (110.3–329.0)	285.0 (194.0–507.0)	0.589
Ferritin (ng/mL)	200.5 (147.2–388.8)	236.0 (63.6–467.0)	217.5 (119.0–361.8)	250.0 (131.3–389.5)	0.998
25-hydroxy vitamin D (ng/mL)	31.9 (24.2–43.1)	24.3 (19.6–27.1)	23.8 (20.7–31.3)	19.4 (17.9–27.0)	0.099
Na (mmol/L)	138.0 ± 3.0	137.8 ± 2.5	138.5 ± 2.7	138.2 ± 3.8	0.876
K (mmol/L)	4.6 ± 0.6	5.1 ± 0.8	4.8 ± 0.8	4.7 ± 0.7	0.085
Ca (mmol/L)	2.3 ± 0.2	2.4 ± 0.2	2.3 ± 0.3	2.2 ± 0.3	0.271
P (mmol/L)	2.0 ± 0.7	2.3 ± 0.6	1.9 ± 0.7	2.3 ± 0.6	0.036
**Volume status**
OH (L)	1.3 ± 1.2	2.2 ± 1.6	2.5 ± 1.5	4.0 ± 2.1	<0.001
ROH (%)	7.5	11.7	15.5	20.6	<0.001
TBW (L)	39.2 ± 7.7	37.8 ± 7.3	32.6 ± 8.4	38.5 ± 7.5	0.006
ECW (L)	17.4 ± 3.0	17.5 ± 3.4	15.7 ± 3.7	18.9 ± 3.6	0.011
ICW (L)	21.8 ± 5.0	20.3 ± 4.4	16.8 ± 5.1	19.6 ± 4.4	0.001
E/I	0.81 ± 0.1	0.87 ± 0.1	0.96 ± 0.1	0.98 ± 0.2	<0.001
BCM	27.3 ± 8.4	24.5 ± 8.6	20.2 ± 8.8	24.2 ± 7.3	0.018
**Echocardiographic examination**
LVMI (g/m^2^)	106.3 ± 23.8	115.1 ± 25.6	122.3 ± 25.7	144.5 ± 42.8	<0.001
LVEF (%)	64.5	64.4	64.1	56.1	<0.001
LAD (mm)	37.1 ± 3.2	40.9 ± 5.0	41.2 ± 4.1	43.9 ± 6.8	<0.001
LVEDD (mm)	46.9 ± 5.2	48.7 ± 4.4	47.9 ± 5.9	51.8 ± 8.2	0.023
LVESD (mm)	30.1 ± 3.4	31.3 ± 3.3	31.0 ± 4.7	36.9 ± 9.5	<0.001
IVST (mm)	10.8 ± 1.6	11.0 ± 1.5	11.2 ± 1.6	11.8 ± 1.6	0.113
PWT (mm)	10.3 ± 1.5	10.3 ± 1.2	10.9 ± 1.6	11.2 ± 1.6	0.049

*Data are presented as means ± SDs or median (interquartile range) for continuous variables and n (%) for categorical variables. NT-pro BNP, N-terminal pro B-type natriuretic peptide; BMI, body mass index; BSA, body surface area; SBP, systolic blood pressure; DBP, diastolic blood pressure; MAP, mean arterial pressure; HR, heart rate; Hb, hemoglobin; HCT, hematocrit; Alb, albumin; Scr, serum creatinine; β_2_MB, β_2_ microglobulin; iPTH, parathyroid hormone; K, potassium; Ca, calcium; P, phosphorus; OH, overhydration; ROH, relative overhydration, ECW, extracellular water; ICW, intracellular water; E/I, extracellular water/intracellular water; BCM, body cell mass; LVMI, left ventricular mass index; LVEF, left ventricular ejection fraction; LAD, left atrial diameter; LVEDD, left ventricular end diastolic diameter; LVESD, left ventricular end systolic diameter; IVST, interventricular septum thickness; PWT, left ventricular posterior wall thickness*.

### Overhydration Risk Factors in Hemodialysis Patients

As illustrated in Table 2, Pearson's simple linear regression analysis showed that OH was associated with Lg (NT-pro BNP) (*r* = 0.467, *P* <0.001). Additionally, OH was positively correlated with height (*r* = 0.294, *P* = 0.001), weight (*r* = 0.206, *P* = 0.019), MAP (*r* = 0.338, *P* <0.001), LVMI (*r* = 0.219, *P* = 0.016), and LAD (*r* = 0.281, *P* = 0.002), but negatively correlated with gender (*r* = −0.221, *P* = 0.012), Hb (*r* = −0.327, *P* <0.001), Alb (*r* = −0.186, *P* = 0.047), SCr (*r* = −0.190, *P* = 0.042), and LVEF (*r* = −0.242, *P* = 0.008).

**Table 2:** Factors associated with the overhydration in patients on hemodialysis.

**Variables**	**Simple linear regression analysis correlation coefficient (*r*)**	***P-*value**	**Stepwise multiple linear regression analysis standardized coefficient (ß)^a^**	***P*-value**
NT-pro BNP (Lg-transformed)	0.467	<0.001	0.217	0.032
Sex	−0.221	0.012		
Age (years)	0.145	0.100	0.249	0.012
Height (cm)	0.294	0.001	0.309	0.008
Weight (kg)	0.206	0.019		
MAP (mmHg)	0.338	<0.001	0.196	0.031
Hb (g/L)	−0.327	<0.001	−0.243	0.007
Alb (g/L)	−0.186	0.047		
SCr (μmol/L)	−0.190	0.042		
LVMI (g/m^2^)	0.219	0.016	0.232	0.017
LVEF (%)	−0.242	0.008		

*NT-pro BNP, N-terminal pro B-type natriuretic peptide; SBP, systolic blood pressure; DBP, diastolic blood pressure; MAP, mean arterial pressure; Hb, hemoglobin; Alb, albumin; Scr, serum creatinine; LVMI, left ventricular mass index; LVEF, left ventricular ejection fraction*.

a *In the stepwise multiple regression model, the overhydration value is the dependent variable. Adjusted R^2^ = 0.505. Sex, weight, albumin, Scr, and LVEF were removed by stepwise elimination*.

Stepwise multiple linear regression analysis showed that the OH value was negatively correlated with Hb (β = −0.243, *P* = 0.007) and positively related to Lg (NT-pro BNP) (β = 0.217, *P* = 0.032), age (β = 0.249, *P* = 0.012), height (β = 0.309, *P* = 0.008), MAP (β = 0.196, *P* = 0.031), and LVMI (β = 0.232, *P* = 0.017). Lg (NT-pro BNP) was independently correlated with OH after adjusting for several potentially confounding variables.

### Association With Volume Overload and NT-Pro BNP

In the subgroup of patients with LVEF ≥ 60%, the association of Lg (NT-pro BNP) with OH persisted (β = 0.236, *P* = 0.044) after adjusting for variables such as sex, age, height, weight, MAP, Hb, Alb, and Scr. However, NT-pro BNP was independently positively related to OH in the group with EF <60% (β = 0.516, *P* = 0.032) (Table 3).

**Table 3:** Association between NT-pro BNP with overhydration in hemodialysis patients with LVEF ≥ 60% and LVEF <60%.

**Variables**	**LVEF** **≥** **60%**		**LVEF** **<** **60%**	
	**Stepwise multiple linear regression analysis standardized coefficient (ß)^a^**	***P-*value**	**Stepwise multiple linear regression analysis standardized coefficient (ß)^b^**	***P*-value**
NT-pro BNP (Lg-transformed)	0.236	0.044	0.516	0.032
Height (cm)			0.689	0.006
MAP (mmHg)	0.238	0.030		

*NT-pro BNP, N-terminal pro B-type natriuretic peptide; MAP, mean arterial pressure; LVEF, left ventricular ejection fraction*.

a *In the stepwise multiple regression model, the overhydration value is the dependent variable. Adjusted R^2^= 0.608. Sex, age, height, weight, Hb, Alb, and Scr were removed by stepwise elimination*.

b *In the stepwise multiple regression model, the overhydration value is the dependent variable. Adjusted R^2^= 0.898. Sex, age, weight, MAP, Hb, Alb, and Scr were removed by stepwise elimination*.

As shown in Table 4, multiple logistic regression analysis showed that each SD increase in Lg (NT-pro BNP) entailed a higher risk of suffering from volume overload (OR = 8.0; 95% CI: 3.316–19.425) in the unadjusted model. When we adjusted for some confounders in the model, each SD increase in Lg (NT-pro BNP) (that is, 0.55) was associated with 6.0 times risk of volume overload (OR = 6.0; 95% CI: 1.971–18.456). However, in the subgroup of patients with LVEF ≥ 60%, we obtained the same result; each SD increase in Lg (NT-pro BNP) (that is, 0.51) was correlated with a higher risk of volume overload (OR = 8.1; 95% CI: 1.943–33.573) in the fully adjusted model. However, Lg (NT-pro BNP) lost statistical significance for volume overload in the group with LVEF <60% (*P* > 0.05).

**Table 4:** Multiple logistic regression analysis on the association of the presence of overhydration with NT-pro BNP.

**Variable**	**OR (95% CI)**	***P*-value**	**OR (95% CI)**	***P*-value**
	**All patients (*****n*** **=** **129)**		**LVEF** **≥** **60% patients (*****n*** **=** **95)**	
**NT-pro BNP (lg-transformed)**				
Unadjusted	8.025 (3.316–19.425)	<0.001	11.816 (3.588–38.916)	<0.001
Adjusted	7.223 (2.622–19.892)	<0.001	8.213 (2.238–30.144)	0.002
Fully adjusted	6.031 (1.971–18.456)	0.002	8.077 (1.943–33.573)	0.004

*NT-pro BNP, N-terminal pro B-type natriuretic peptide; LVEF, left ventricular ejection fraction*.

*In the model, adjusted for age, sex, height, weight, and MAP, fully adjusted for Hb, Alb, and Scr*.

**Figure 1:**
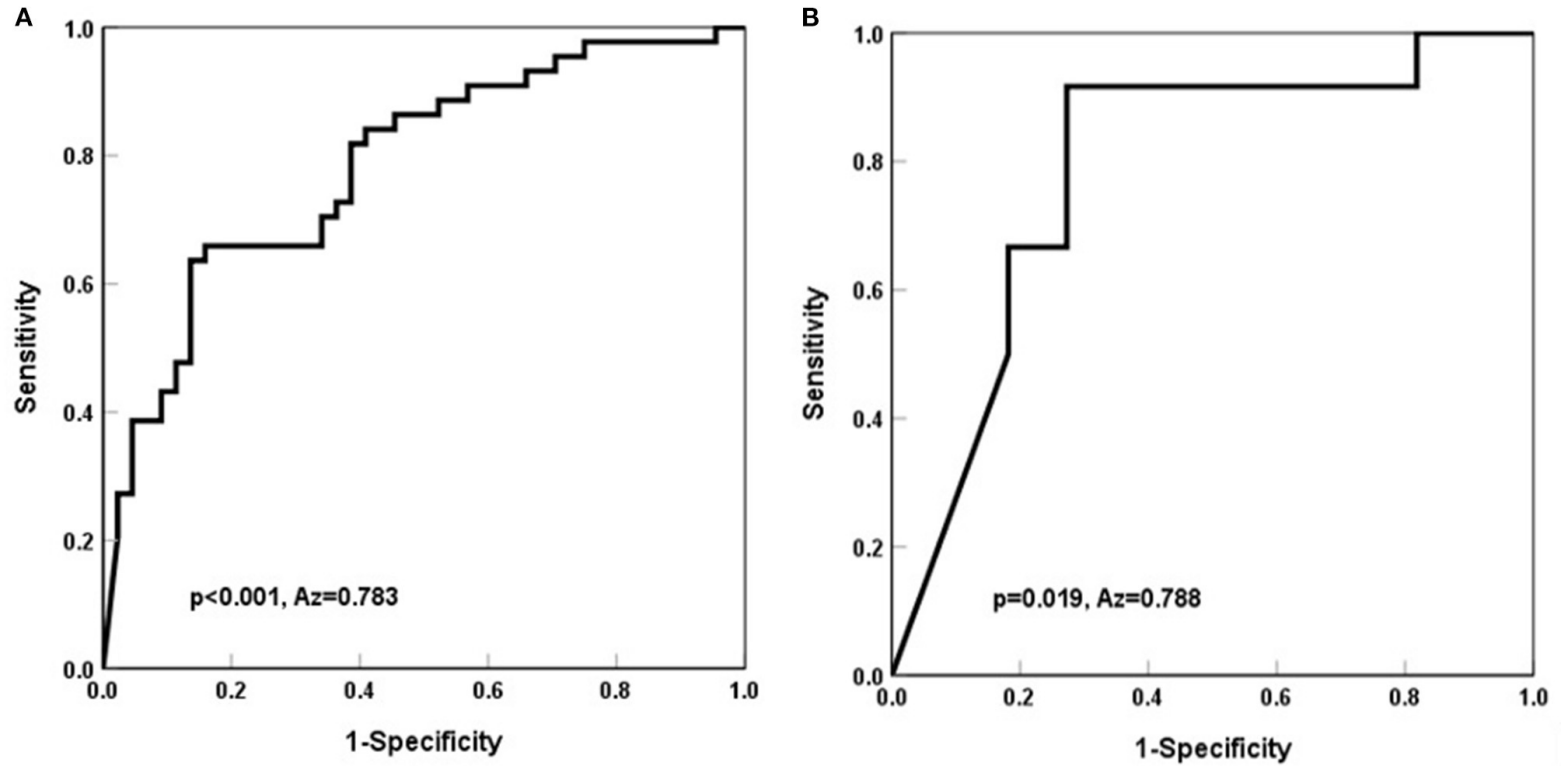
Receiver operating characteristic curve for predictive factor (NT-pro BNP) associated with volume overload in hemodialysis patients. **(A)** In the group with LVEF ≥ 60%, the cutoff value was 5,741.5 pg/mL, the sensitivity was 63.6%, and the specificity was 86.4%. The AUC was 0.783. AUC, area under the curve. **(B)** In the group with EF <60%, the cutoff value was 15,617.5 pg/mL, the sensitivity was 91.7%, and the specificity was 72.7%. The AUC was 0.788. AUC, area under the curve.

Based on the cutoff value, we built the receiver operating characteristic (ROC) curve to determine the potential utility of NT-pro BNP for evaluating volume overload in the LVEF ≥ 60% and LVEF <60% groups. In the subgroup of patients with LVEF ≥ 60%, the area under the curve was 0.783 [95% CI (0.688–0.879), *P* <0.001], the cutoff value of NT-pro BNP was 5,741.5 pg/mL, the sensitivity was 63.6%, and the specificity was 86.4%. However, in the LVEF <60% subgroup, the area under the curve was 0.788 [95% CI (0.586–0.989), *P* <0.001], the cutoff value for NT-pro BNP was 15,617.5 pg/mL, the sensitivity was 91.7%, and the specificity was 72.7% in the LVEF <60% group (Figure 1).

## Discussion

Our results indicate that NT-pro BNP level was correlated with overhydration. Each SD increase in Lg (NT-pro BNP) was associated with a 6-fold risk of volume overload. The ROC curve showed that the cutoff value of NT-pro BNP was 5,741.5 pg/mL for evaluating volume overload in hemodialysis patients of LVEF ≥ 60%, and which of NT-pro BNP was 15,617.5 pg/mL for LVEF <60% dialysis patients, respectively.

Early diagnosis and treatment of volume overload is crucial in the improvement of patients' quality of life. In clinical practice, patients' hydration status is determined based on physical findings, such as weight gain, peripheral edema, blood pressure, and pulmonary rales. However, this method is subjective and is associated with poor diagnostic accuracy as these do not accurately reflect the hydration status of patients. In recent years, much efforts have been allocated into identifying accurate and objective methods for evaluating hydration status (8).

Bioimpedance spectroscopy is the most widely used, noninvasive method of analyzing the body's fluid distribution (21). Some studies have shown consistencies between the volume status measured by bioimpedance spectroscopy and the gold standard methods (deuterium or tritium dilution) (22). Further, studies have shown that volume overload, as measured by the BCM, could act as an independent predictor of mortality (23, 24).

In our study, we demonstrated an association between NT-Pro BNP and several variables of volume status. With an increase in NT-pro BNP, the value of OH and the ratio of OH and ECW/ICW increased. The value of overhydration was 2.68 ± 0.19 L and the incidence rate of volume overload was 53.9% in our hemodialysis patients who had minimal peripheral edema. These findings are similar to those of Madsen et al., who reported that NT-pro BNP levels were markedly elevated in 109 hemodialysis patients (25). Stenberg et al. reported that pre-dialysis NT-pro BNP was correlated with ECW/TBW and ROH (26). Additionally, we noted that the higher quartile NT-pro BNP group had higher SBP, LAD, and LVMI. Similar associations of LVMI were found for NT-pro BNP in both hemodialysis and PD patients (27). These results show that high volume load led to persistent hypertension, which eventually caused left atrial and ventricular hypertrophy.

Studies have shown that 55–65% of serum NT-pro BNP is cleared by the renal tissue, and this function is retained even in situations of moderate kidney dysfunction (28). Additionally, the concentration of NT-pro BNP increases as renal function decreases (29). Higher serum NT-pro BNP concentration in CKD patients was associated with the degree of subsequent kidney function decline (30). Studies have demonstrated a significant association between NPs and cardiovascular diseases or various indicators of cardiac structure and function, such as LVMI, LVEF, and fractional shortening (31, 32). Therefore, BNP and NT-pro BNP have been established as biomarkers of heart failure in the general population and in CKD patients (33, 34). However, a study by Lei et al. demonstrated that NT-pro BNP could not be used as a marker of improvement of heart failure in patients with renal failure who were undergoing hemodialysis (35). Moreover, little is known about the utility of NT-pro BNP as an indicator of volume status in the presence of both cardiac structural and functional change, and volume overload.

In the present study, we found that LVEF decreased gradually in the higher quartile NT-pro BNP group. Similarly, the value of overhydration and the proportion of volume overload were higher in the LVEF <60% group than in the LVEF ≥ 60% group (Supplementary Table 1). These results demonstrate that decreased cardiac ejection function, water-sodium retention, and circulatory capacity overload contribute to NT-pro BNP release from the myocardium making it a viable indicator for cardiac function and volume status.

In addition, we found that in the subgroup of EF <60%, NT-pro BNP was correlated with overhydration after adjustments for all potential confounders. When NT-pro BNP was above the cut-off value, volume overload was present in patients. The results demonstrate an association between NT-pro BNP and overhydration, despite the presence of reduced cardiac ejection fraction.

This study has a few limitations. First, the study was a cross-sectional design, some potential predictive variables and outcome variables were acquired almost simultaneously, and information about causality could not be provided. Therefore, long-term and longitudinal studies should be considered in the future. Second, this study involved a relatively small size and the proportion of patients with reduced cardiac ejection fraction was less than those who experienced an increase, which might have affected the logistic regression analysis of the correlation between NT-pro BNP and volume overload in the group with EF <60%. Hence, a larger sample size of patient is required in further studies.

## Conclusions

This cross-sectional study demonstrated that NT-pro BNP is an effective marker of volume status in patients with or without reduced ejection fraction undergoing hemodialysis.

## Data Availability Statement

The original contributions presented in the study are included in the article/Supplementary Material, further inquiries can be directed to the corresponding author/s.

## Ethics Statement

The studies involving human participants were reviewed and approved by the Ethics Committee of Zhongshan Hospital, Fudan University. The patients/participants provided their written informed consent to participate in this study.

## Author Contributions

YW and JY conceived and designed the study. XL, XCh, and YZ collected the data. JZ and XCa helped to analyze the data. YW wrote the article. BS and XD reviewed and edited the manuscript. All authors contributed to the article and approved the submitted version.

## Conflict of Interest

The authors declare that the research was conducted in the absence of any commercial or financial relationships that could be construed as a potential conflict of interest.
