# Supplementary material for: Association of N-Terminal Pro-brain Natriuretic Peptide With Volume Status and Cardiac Function in Hemodialysis Patients
Source: Front Cardiovasc Med. 2021 Feb 22;8:646402. doi: 10.3389/fcvm.2021.646402 (PMC7937607; doi:10.3389/fcvm.2021.646402)
Supplement: Supplementary file 1 [file Table_1.docx]

Supplementary Material

**Supplementary Table 1. Baseline characteristics of hemodialysis patients of LVEF≥60% and <60%**

| **Characteristic** | **LVEF≥60%**  **(n=101)** | **LVEF<60%**  **(n=28)** | ***P* Value** |
| --- | --- | --- | --- |
| **Age (years)** | 59.9±12.6 | 58.0±15.6 | 0.522 |
| **Sex (men, %)** | 54.7 | 69.2 | 0.185 |
| **HD duration (months)** | 58.0 (24.7-96.1) | 36.3 (20.4-69.6) | 0.090 |
| **BMI (kg/m^2^)** | 22.4±3.6 | 23.3±4.0 | 0.265 |
| **BSA (m^2^)** | 1.64±0.2 | 1.73±0.2 | **0.038** |
| **SBP (mmHg)** | 143±23 | 149±24 | 0.286 |
| **DBP (mmHg)** | 79±15 | 80±17 | 0.611 |
| **MAP (mmHg)** | 100±16 | 103±18 | 0.407 |
| **HR (bpm)** | 76±11 | 77±11 | 0.648 |
| **Laboratory parameters** |  |  |  |
| Hb (g/L) | 113±14 | 107±18 | 0.135 |
| HCT (%) | 36±5 | 34±6 | 0.114 |
| Alb (g/L) | 40±3 | 38±9 | 0.326 |
| Cr (μmol/L) | 988±285 | 940±321 | 0.488 |
| β_2_MB (mg/L) | 34.9±8.5 | 33.3±10.9 | 0.454 |
| iPTH (pg/mL) | 233.5 (162.8-432.3) | 279.5 (148.8-321.0) | 0.732 |
| Ferritin (ng/mL) | 220.0 (111.5-391.3) | 160.5 (108.0-355.5) | 0.494 |
| 25-hydroxy vitamin D (ng/mL) | 35.1 (21.1-31.9) | 20.7 (18.0-25.5) | 0.055 |
| Na (mmol/L) | 138.1±3.1 | 138.0±2.8 | 0.800 |
| K (mmol/L) | 4.9±0.7 | 4.7±0.8 | 0.351 |
| Ca (mmol/L) | 2.3±0.2 | 2.2±0.3 | 0.080 |
| P (mmol/L) | 2.2±0.6 | 2.1±0.7 | 0.619 |
| NT-Pro BNP (pg/mL) | 4006.5 (1865.3-8632.0) | 26303.0 (4992-35000.0) | **<0.001** |
| **Volume status** |  |  |  |
| OH (L) | 2.5±2.1 | 3.2±2.3 | 0.129 |
| ROH (%) | 52.6 | 53.8 | 0.912 |
| TBW (L) | 36.6±8.3 | 39.6±7.8 | 0.102 |
| ECW (L) | 17.1±3.9 | 18.9±3.5 | **0.043** |
| ICW (L) | 19.5±4.9 | 20.8±5.0 | 0.242 |
| E/I | 0.90±0.1 | 0.93±0.2 | 0.249 |
| BCM | 24.9±8.4 | 25.2±8.5 | 0.534 |

| **Echocardiographic examination** |
| --- |

| LVMI (g/m^2^) | 112.5±26.1 | 152.0±37.2 | **<0.001** |  |
| --- | --- | --- | --- | --- |
| LAD (mm) | 39.6±4.7 | 45.4±5.3 | **<0.001** | |
| LVEDD (mm) | 47.2±4.9 | 55.1±6.3 | **<0.001** | |
| LVESD (mm) | 30.0±3.1 | 41.0±6.9 | **<0.001** | |
| IVST (mm) | 11.0±1.6 | 11.7±1.6 | 0.062 | |
| PWT (mm) | 10.4±1.4 | 11.4±1.7 | **0.003** | |

Data are presented as means ± SDs or median (interquartile range) for continuous variables as appropriate and n (%) for categorical variables. NT-pro BNP, N-terminal pro B type natriuretic peptide; BMI, body mass index; BSA, body surface area; SBP, systolic blood pressure; DBP, diastolic blood pressure; MAP, mean arterial pressure; HR, heart rate; Hb, hemoglobin; HCT, hematocrit; Alb, albumin; Scr, serum creatinine; β_2_MB, β_2_ microglobulin; iPTH, parathyroid hormone; K, potassium; Ca, calcium; P, phosphorus; OH, overhydration; ROH, relative overhydration, ECW, extracellular water; ICW, intracellular water; E/I, extracellular water/intracellular water; BCM, body cell mass; LVMI, left ventricular mass index; EF, left ventricular ejection fraction; LAD, left atrial diameter; LVEDD, left ventricular end diastolic diameter; LVESD, left ventricular end systolic diameter; IVST, interventricular septum thickness; PWT, left ventricular posterior wall thickness.
